# Poor level of knowledge on elderly care despite positive attitude among nursing students in Zanzibar Island: findings from a cross-sectional study

**DOI:** 10.1186/s12912-020-00488-w

**Published:** 2020-10-09

**Authors:** Arafa A. Muhsin, Mariam J. Munyogwa, Stephen M. Kibusi, Saada A. Seif

**Affiliations:** 1 Department of Nursing and Midwifery, University of Dodoma, Dodoma, Tanzania; 2 Department of Nursing and Midwifery, Zanzibar University, Zanzibar, Tanzania; 3 Department of Public Health, University of Dodoma, Dodoma, Tanzania

**Keywords:** Elderly care, Nursing students, Nursing knowledge, Nursing attitudes

## Abstract

**Background:**

It is estimated by the year 2050, 80% of the global elderly population will be from the low-and middle income countries. Elderly care requires health workers with skills associated with an understanding of the biological, psychological, social and cultural theories related to aging. Nurses with better knowledge, skills and positive attitudes towards elderly care are highly needed and critically important for better healthcare and wellbeing of the elderly population. Therefore the objective of this study was to assess the level of knowledge and attitude of nursing students towards elderly care in Zanzibar Island.

**Methods:**

A cross-sectional study was conducted in Zanzibar involving three out of five nursing training institutions. Participants were selected by systematic random sampling. Facts on Aging Quiz 2 and Kogan’s Attitudes Toward Old People scale were used to assess the level of knowledge and attitude towards elderly care among the students respectively. Simple and multivariable logistic regressions were applied to determine the predictors of knowledge and attitude among the participants.

**Results:**

A total of 393 students participated in this study. Only 17% (69) of the participants had good level of knowledge and about 67.9% (267) had positive attitude towards elderly care. Living in an extended family and with an elderly person at home were both associated with good level of knowledge and positive attitude towards elderly care. Furthermore, living in a rural area (adjusted odds ratio = 2.23; 95% confidence interval: 1.22, 4.10) and studying at public institution (adjusted odds ratio = 2.59; 95% confidence interval: 1.41, 4.63) were associated with positive attitude towards elderly care.

**Conclusion:**

This study has shown that the majority of nursing students in Zanzibar have positive attitude but poor level of knowledge towards elderly care. The current findings have demonstrated that past experience with an elderly person can help in influencing good knowledge and shaping positive attitudes towards elderly care. Low level of knowledge shown in the study suggests for further research on adequacy of nursing curriculum and/or its implementation.

## Background

The healthcare system is currently observing a new emerging context of elderly people’s greater demand for health care services [1]. The population of elderly is increasing dramatically in both proportion and absolute number across the world [2] and the rate is greater in lower and middle-income countries (LMICs) compared to high-income countries. It is estimated that by the year 2015, 80% of global elderly population will be living in LMICs [3, 4]. In sub-Saharan Africa (SSA), elderly population is expected to triple within 35 years period from 46 million in 2015 to 157 million in 2050 [2].

Elderly age is associated with changes that constitute a gradual decrease in physiological reserves, an increased risk of chronic diseases, and a general decline in the capacity of the individual [2, 5]. Generally from 60 years old, the major burdens of disability and death arise from age related losses in hearing, seeing and moving as well as noncommunicable diseases including heart disease, stroke, chronic respiratory disorders, cancer and dementia [6]. Therefore, healthy ageing requires health systems that will be able to prevent chronic conditions or ensure early detection and control, of the decline in function capacity and managing of advanced chronic conditions [2]. Availability of these services are important in helping people navigate the health systems and collect the resources that will enable them to deal with the health issues that often arise in older age [7]. 

Elderly care requires health workers with specific skills associated with an understanding of the biological, psychological, social and cultural theories related to aging [2]. However, many health professionals are often not adequately prepared to deal with the healthcare needs of older adults because many existing training curriculum were developed by twentieth century and had focused much on management and treatment of acute infectious diseases which were the world’s most prevailing health problems [2]. Previous studies have shown that adequately prepared nurses with better knowledge, skills and positive attitude towards older adults can improve patient outcomes such as reduced hospital length of stay, reduced readmission rates and increased patient and family satisfaction [8, 9]. But little is known about the level of knowledge and attitude of nurses in provision of elderly care in Tanzania. Therefore the objective of this study was to assess the level of knowledge and attitude of nursing students toward elderly care in Zanzibar. The findings from this study form a basis of identifying the competence gaps to inform curriculum implementers and foster for the discussion among nurse educators and associated stakeholders on adequacy of the curriculum in preparing student nurses to provide the quality elderly care in the country.

## Methods

### Study setting

This study was conducted in Zanzibar Island, Tanzania. Zanzibar is one of the Indian Ocean islands. Administratively, it comprises of two main islands; Unguja and Pemba. According to the 2012 Tanzania housing and population census, the island has approximately total population of 1.3 m with 2.8% annual growth rate and 5.1 average household size. The largest ethnic group is the Swahili speaking people.

Regarding elderly welfare, in Zanzibar there are three registered elderly care centres; Two centres are government owned and one centre is a faith based hospital. In all the centres, daily service providers are mainly nurses and social workers. Further, since after the Zanzibar Revolution to date, the Government of Zanzibar provides free medical services to all people aged 60 years and above. Furthermore, from 2016 the government of Zanzibar introduced monthly allowance to all older people aged 60 years and above.

Until June 2019, Zanzibar has a total of five registered nursing training institutions namely; Zanzibar University (ZU), State University of Zanzibar (SUZA), Pemba School of Health, Zanzibar School of Health and Mwenge Community College. Among five, four institutions are owned by private sector and the remaining one is a public institution. Presently, all the institutions train students at diploma level and have approximately a total of 1232 students. The diploma in nursing training is a 3 years programme offered under the supervision of Zanzibar Nursing and Midwifery Council (ZNMC).

### Study design and study population

This study was a cross sectional study, conducted from April 2019 to June 2019. The study population was diploma nursing students. Students in the first year of study were excluded from the study.

### Sample size and sampling procedures

Sample size was calculated using the formula; $n=\frac{z^2p\left(1-p\right)}{e^2}$ whereby, n = the minimum required sample size, z = level of confidence (1.96), p = parameter for sample calculation (50%), e = margin of error (0.05). Purposive sampling was used to select the only one public institution and simple random sampling was used to select two out of four private institutions. Systematic random sampling was used to select 393 participants from a list of 509 s and third year registered students obtained from the respective institution administration.

### Data collection and study procedure

Self administered English questionnaire was used to conduct the study. All information from the participant was collected and recorded in a standard questionnaire (see Additional file 1). The questionnaire was divided into four parts; demographic characteristics, student experience on elderly caring, questions on knowledge and statements on attitude towards elderly care. The researcher and research assistants supervised the whole process of questionnaire administration to prevent contamination among students.

Demographic characteristics assessed were age of the student, marital status, sex, type of home residence, year of study, type of family and type of the institution. Regarding student experience on elderly care participants were asked the questions on; living with an elderly at home, experience of caring for older people before joining nursing school, where did they get the experience of caring elderly and frequency of contact with an elderly person before coming to the nursing college. Further, knowledge and attitude of participants towards elderly care were assessed by using standardized tools as follows;

The Facts on Aging Quiz 2 (FAQ 2) was used to test knowledge [10]. The tool consists of 25 true-false statements which measures knowledge related to basic physical, psychological and social facts on aging. Scoring system of student’s knowledge was one point for the correct answer and zero point for incorrect or no answer. The knowledge score for each participant was the sum of the correct answers. The scores ranged from 0 to 25 points. The higher the score indicated greater knowledge on aging and elderly care. The participants’ final scores were classified as follows; 0–12 points = poor knowledge 13–25 points = good knowledge.

Attitude toward elderly care was assessed by using Attitudes Toward Old People (ATOP) scale [11]. The ATOP scale was a 34-item Likert-type instrument consisting of 17 matched positive and negative item pairs of attitudinal statements about the elderly. It was a 5 point Likert-type scale ranging from 1 = strongly disagree, 2 = disagree, 3 = neutral, 4 = agree and 5 = strongly agree. Overall scores ranged from 34 to 170 points whereby the higher score indicated the positive the attitude and vice versa. The final participants’ scores were then classified as follows; 34–84 points = negative attitudes and 85–170 points = positive attitudes.

### Data analysis

Data were analyzed using the Statistical Package for the Social Sciences (SPSS) version 22 for Windows computer program (SPSS Inc. Chicago). Preliminary data analysis included descriptive statistics, i.e. means and standard deviations for continuous variables and frequencies and percentages for categorical variables which describe the population characteristics. Further, Chi-square test was conducted to compare the proportions of knowledge and attitude with different demographic variables of participants. Furthermore, all variables with a *p*-value of < 0.05 were considered to be significant and entered in simple and multivariable logistic regression to determine the predictors of knowledge level and attitude towards care of elderly among the participants.

## Results

### General characteristics of the study participants

General characteristics of the study participants are shown in Table 1. A total of 393 participants were involved in this study. About 69.7% (274) of the participants were female. The mean age of the participants was 22.83 ± 1.95 years. About 54.7% (215) were in second year of the study. Participants from urban areas constitute 66.7% (262). Most of the participants 54.5% (214) grew up from extended families. More than half of the participants reported to be living with an elderly person at their homes currently.

**Table 1:** General characteristics of the study participants (*N* = 393)

Participants’ characteristics	n	%
Sex		
Male	119	30.3
Female	274	69.7
Age		
18–24	341	86.8
25–33	52	13.2
Type of the Institution		
Public	140	35.6
Private	253	64.4
Year of study		
Second year	215	54.7
Third year	178	45.3
Marital Status		
Never married	358	91.1
Ever married	35	8.9
Home residence		
Rural	131	33.3
Urban	262	66.7
Type of family		
Nuclear family	179	45.5
Extended family	214	54.5
Currently living with an elderly person at home		
Yes	210	53.4
No	183	46.5

### Distribution and predictors of knowledge level on elderly care among the study participants

Table 2 shows the distribution and predictors of knowledge on elderly care among the study participants. Generally, only 17.6% (69) of all the participants had good knowledge on elderly care. The results of chi-square test showed that, the level of knowledge on elderly care among participants differed significantly by type of institution, type of family and currently being living with elderly at home. It was shown that, proportion of students with good knowledge was significantly higher among the participants at private institutions (20.6%, *n* = 52; *p* < 0.05), those from extended families (29.4%, *n* = 63; *p* < 0.0001) and the participants living with an elderly person at home (31.0%, *n* = 65; *p* < 0.0001).

**Table 2:** Distribution and predictors of knowledge on elderly care among the study participants

Variable	Good knowledge (%) ^**#**^	OR (95% CI)	***p***-value	AOR (95% CI	***p***-value
Sex			0.261	–	–
Male	17 (14.3)*	1		–	–
Female	52 (19.0)	1.4 (0.78, 2.55)		–	–
Age			0.658	–	–
18–24	61 (17.9)*	1.19 (0.54, 2.67)		–	–
25–33	8 (15.4)	1		–	
Type of the Institution			0.036		0.124
Public	17 (12.2)**	1		1	
Private	52 (20.6)	1.87 (1.04, 3.38)		1.66 (0.87, 3.17)	
Year of study			0.547	–	–
Second year	31 (17.4)*	1		–	–
Third year	38 (17.7)	1.02 (0.60, 1.72)			–
Marital Status			0.186		0.461
Never married	60 (16.8)*	1		1	
Married	9 (25.7)	1.72 (0.77, 3.85)		1.41 (0.57, 3.49)	
Home residence			0.574	–	–
Rural	25 (19.1)*	1.17 (0.67, 2.01)		–	–
Urban	44 (16.8)	1		–	–
Type of family			< 0.0001		0.001
Nuclear family	6 (3.4)***	1		1	
Extended family	63 (29.4)	12.03 (5.06, 28.58)		4.73 (1.85, 12.11)	
Currently living with an elderly person at home			< 0.0001		< 0.0001
No	4 (2.2)***	1		1	
Yes	65 (31.0)	20.06 (7.14, 5.37)		9.04 (3.00, 27.21)	

^#^Differences in prevalence was assessed by chi-square test and the *p*-value obtained were as follows; *** p < 0.0001, ** p < 0.05, *p ≥ 0.05

After applying multivariable logistic regression, predictors of good level of knowledge were; experience of living with an elderly person at home and living from an extended family. Results showed that the odds of having good knowledge was nine times greater among the participants living with an elderly person at home (AOR = 9.04; 95% CI: 3.00, 27.21) than those without an elderly person at home. Furthermore participants who come from an extended family had greater odds of having good knowledge compared to those who came from nuclear family (AOR = 4.73; 95% CI: 1.85, 12.11) (Table 2).

### Distribution and predictors of attitude towards elderly care among the study participants

Table 3 shows the distribution and predictors of attitude towards elderly care among the study participants. Generally, most 267 (67.9%) of the participants had positive attitudes towards elderly. The results of chi-square test showed that, attitudes towards elderly differed significantly by type of institution, year of study, home residence, type of family and currently living with an elderly person at home. It was shown that, the proportion of students with positive attitude towards elderly care was significantly higher among the participants at public institutions (76.4%, *n* = 107; *p* < 0.05); the participants from rural residence (83.2%, *n* = 109; *p* < 0.0001); the participants from extended families (85.0%, *n* = 189; *p* < 0.0001) and among the participants living with an elderly at home (87.1%, *n* = 183; *p* < 0.0001).

**Table 3:** Distribution and predictors of attitudes towards elderly care among the study participants

Variable	Positive attitudes (%)^**#**^	OR (95% CI)	***p***-value	AOR (95% CI	***p*** - value
Sex			0.613	–	–
Male	83 (69.7)***	1		–	–
Female	184 (67.2)	0.89 (0.56,1.41)		–	–
Age			0.830	–	–
18–24	231 (67.7)***	1		–	–
25–33	36 (69.2)	1.07 (0.57, 2.601)		–	
Type of the Institution			0.008		0.001
Public	107 (76.4)**	1.89 (1.18, 3.00)		2.59 (1.45, 4.63)	
Private	160 (63.2)	1		1	
Year of study			0.053		0.211
Second year	155 (72.1)**	1.52 (0.99, 2.33)		1.41 (0.83, 2.39)	–
Third year	112 (62.9)	1		1	–
Marital Status			0.643		
Never married	242 (67.6)***	1			
Married	25 (71.4)	1.19 (0.56, 2.58)			
Home residence			< 0.0001		0.010
Rural	109 (83.2)*	3.26 (1.94, 5.49)		2.23 (1.22, 4.10)	
Urban	158 (60.3)	1		1	
Type of family			< 0.0001		< 0.0001
Nuclear family	78 (43.6)*	1		1	
Extended family	189 (88.3)	9.79 (5.87, 16.32)		4.34 (2.33, 8.09)	
Currently living with an elderly person at home			< 0.0001		< 0.0001
No	84 (45.9)*	1		1	
Yes	183 (87.1)	7.99 (4.86, 13.14)		3.65 (1.96, 6.78)	

^#^Differences in prevalence was assessed by chi-square test and the *p*-value obtained were as follows;

* p < 0.0001, ** p < 0.05, *** p ≥ 0.05

After applying multivariable logistic regression; family type, experience of living with an elderly person at home, rural residence and studying at public institution were associated with positive attitudes towards elderly care. The results showed that, participants living from extended family had four times odds of having positive attitudes on elderly care (AOR = 4.34; 95% CI: 2.33, 8.09) compared to the matching part. The odds of having positive attitude was greater by three and more times among the participants living with an elderly person at home (AOR = 3.65; 95% CI: 1.96, 6.78, *p* < 0.0001) than the matching part. Participants from rural residence (AOR = 2.23; 95% CI: 1.22, 4.10) and those studying at public institutions (AOR = 2.59; 95% CI: 1.41, 4.63) had more than two odds of having positive attitude compared to their matching parts (*p* < 0.0001) (Table 3).

## Discussion

This study generally found poor knowledge and positive attitude towards elderly care among nursing students in Zanzibar. Students living with an elderly person at home and those from extended family were more likely to have good knowledge and positive attitude towards elderly. Further, students at public institutions and those from rural areas were associated with positive attitudes.

This study found that, majority of students had poor knowledge on basic knowledge for physical, psychological and social changing of elderly people. The current finding is similar with findings from other cross-sectional studies conducted among nursing students in Egypt [12] and China [13] which also demonstrated poor knowledge on elderly care among nursing students. Further, the study done in Egypt demonstrated that, insufficient gerontological courses and lack of clinical exposure among nursing students contribute to poor knowledge on elderly care [12]. In the current study, the reasons for the poor knowledge were not literary clear because the study involved second and third year diploma in nursing students who were expected to be already taught gerontological courses and started clinical practices during their first year and first semester of second year of study respectively. Therefore, this finding highlights the need to do the assessment on the adequacy of the current curriculum and its implementation plan to provide quality elderly care which were not assessed during this study.

Contrary to this finding, a cross-sectional study conducted in Nigeria demonstrated higher proportion of nursing students with good knowledge on elderly care [14]. Unlike our study which involved only diploma students, the study in Nigeria involved both diploma and bachelor degree nursing students. Compared to this study, the bachelor degree students in Nigeria might have influenced the higher proportion of students with good knowledge.

Interestingly, this study found that, being currently living in an extended family and having an elderly member at home were significantly associated with good knowledge on elderly care among the participants. Experience gained while taking care of an elderly and also living with other relatives at home can possibly explain this finding. In most SSA countries, it is the customary responsibility of the young ones to provide assistance to their older relatives in various aspects. Such care may include; helping an elderly member to perform activities of daily living, preventing falls and abuse among elderly and ensuring their body hygiene. It is also the duty of the family members to assist an elderly member on other simple healthcare related tasks such as administering their medication, accompanying elderly to their scheduled doctor’s appointment and taking of vital signs such as measuring blood glucose and blood pressure [15–17]. Further, the family members also play a role in providing physical, emotional, psychosocial and sometimes financial assistance to older relatives who cannot care for themselves [18].

In this study, majority of the students had positive attitudes towards elderly care. This is a promising finding as attitude is very crucial in influencing nursing professionals to work with older people [14]. Previous findings had revealed that nursing students with positive attitudes were more willing to work with elderly population than those with negative attitudes [13, 19, 20]. The result of positive attitude in this study may be attributed to the cultural and societal values of low and middle-income countries which accord older adults with high respect [21].

In most African countries, it is a responsibility and pride to look after older relatives [15]. The positive attitude found in this study is in agreement with the findings from the previous cross-sectional studies which reported high prevalence of positive attitude towards older adults among nursing students at the university college [12, 22]. Contrary to our findings, a cross-sectional study conducted among working nurses in Iran found that majority of them had negative attitudes towards elderly care [23]. Compared to the previous study, the positive attitude observed in the current study may be explained with the fact that our study population was students and young population without working experience or little experience if any. Studies have demonstrated that negative attitude on elderly care among nurses may be influenced by work related factors such as; low salaries, heavy workload, a boring and unattractive field, lower status of geriatric nurses, difficulty in communicating with geriatric patients and limited care resources in working areas [23, 24]. This finding suggests that, inorder to build and maintain positive attitudes towards elderly care among nurses both training and working environments are indispensable.

Interestingly, in this study it was also demonstrated that, living from extended family and having an elderly member at home were the factors associated with positive attitudes towards elderly among the students. The possible explanation for this finding could be the experience gained after frequently being in contact with an elderly member at their homes [12, 22]. This finding is supported by the finding from cross-sectional study conducted in Iran which shows that attitude scores was higher among nurses with an elderly member at home than those who do not have one [23]. The current finding suggests an emphasis on reinforcing exposure and active involving nursing students with elderly population because this will help to build the interest of nursing students towards elderly care [8, 25]. This will ensure graduate nurses with good attitudes on elderly population and able to provide quality care for the elderly people. Practical training should ensure that nurses receive adequate contact with an elderly during their training.

In this study it was found that students residing from rural areas were more likely to have positive attitudes than those from urban areas. The possible explanation for this finding could be that, students from rural settings are expected to have frequent contact with elderly people compared to those from urban areas. This is because elderly people are mostly living in rural areas. Further, extended family structure where elderly person may be one of the family members is more practiced in rural settings than urban. This finding continues to emphasize the importance of experience on elderly exposure for building positive attitudes towards elderly people among nursing students.

This study has demonstrated that higher proportion of students with positive attitudes were from public institutions as compared to those from private institutions. In Zanzibar all nursing training institutions are under the supervision of the nursing education and practice of Zanzibar Nursing and Midwifery Council. Unfortunately, the current study couldn’t find the possible explanation of this finding from the literature. Therefore we propose for the future scholars to look for the preparedness of these institutions in provision of elderly care training such as nature of clinical training [14], the curriculum [25, 26] and others which weren’t assessed during this study.

## Conclusion

This study has shown that the majority of nursing students have positive attitudes but poor knowledge on elderly care in Zanzibar. Generally, past experience with an elderly person at home and extended family structure were associated with both good knowledge and positive attitude. Further, rural dwellers and public institutions students were significantly associated with positive attitudes. The current findings, emphasizes the importance of reinforcing exposure of nursing students and actively involving them with older adults, during their training. This might ensure graduating nurses with good knowledge and positive attitudes on elderly population thus improve the elderly care and the well-being of older people. Furthermore, future research should consider assessing the adequacy of the nursing curriculum in providing knowledge on elderly care and its implementation plan in order to identify the training gaps.

## Supplementary information

**Additional file 1.** Questionnaire for assessment of knowledge level, attitudes and their predictors towards elderly care among nursing students in Zanzibar. A cross-sectional study. The questionnaire shows questions used to collect information on demographic characteristics, past history on elderly and to assess the knowledge and attitude level towards elderly care among nursing students in Zanzibar.

## Data Availability

The datasets used and/or analysed during the current study are available from the corresponding author without any restriction.

## Abbreviations

LMICs: Low-and middle income countries
SSA: Sub-Saharan Africa
HIV: Human immunodeficiency virus
AIDS: Acquired immunodeficiency syndrome
TB: Tuberculosis
ZU: Zanzibar University
SUZA: State University of Zanzibar
ZNMC: Zanzibar Nurses and Midwives Council
FAQ: Facts on ageing quiz
ATOP: Attitudes Towards Old people
AOR: Adjusted odds ratio
CI: Confidence interval
