# Supplementary material for: Poor level of knowledge on elderly care despite positive attitude among nursing students in Zanzibar Island: findings from a cross-sectional study
Source: BMC Nurs. 2020 Oct 9;19:96. doi: 10.1186/s12912-020-00488-w (PMC7547516; doi:10.1186/s12912-020-00488-w)
Supplement: Supplementary file 1 — Additional file 1. Questionnaire for assessment of knowledge level, attitudes and their predictors towards elderly care among nursing students in Zanzibar. A cross-sectional study. The questionnaire shows questions used to collect information on demographic characteristics, past history on elderly and to assess the knowledge and attitude level towards elderly care among nursing students in Zanzibar. [file 12912_2020_488_MOESM1_ESM.docx]

Questionnaire for assessment of knowledge level, attitudes and their predictors towards elderly care among nursing students in Zanzibar.  A cross-sectional study

Questionnaire Number ……………………………..

Date ……………………………………….

**PART I: Social demographic data**

**Instructions: Please circle the correct answer for you**

1. Your gender
2. Male
3. Female
4. Age …….
5. Name of your Institution?
6. Zanzibar University
7. State University of Zanzibarr
8. Zanzibar School of Health
9. Pemba School of Health
10. Mwenge Community College
11. Type of institution
    1. Public
    2. Private
12. Name of your home residential ward………………………………
13. Type of home residence
14. Rural
15. Urban
16. Year of study?
17. Second year
18. Third year
19. Marital Status
20. Single
21. Married
22. Divorced
23. Widowed
24. With whom did you live with, while growing up?
25. Mother, father and Siblings only
26. Mother, father, grandparents, siblings etc.
27. Grandparents, aunties, uncles and cousins etc
28. Other… Specified ……………………………....

**PART II: Past experience with elderly**

1. Are there any elderly people aged 60+ living in your home?
2. Yes
3. No
4. Did you have any experience of caring older people (60+yrs) before joining this Nursing Course?
5. Yes
6. No
7. If the above answer is yes, where did you experience that care?
8. Caring for older family members
9. Voluntary work
10. Visiting Nursing home
11. Working as a support worker
12. How often do you come in contact with people aged 60 or over?
13. Daily
14. Twice a week
15. Weekly
16. Once a month
17. Never
18. Have you already cared for elderly person age 60+ in your clinical nursing practice?
19. Yes
20. No

**PART II: Knowledge of Aging: Knowledge about the ageing process as measured by Facts on Aging Quiz (25 questions)**

**Please tick appropriate letter (T = true, F = false)**

|  | Questions | **True** | **False** |
| --- | --- | --- | --- |
| 1 | Life expectancy of Tanzanian at age 60+ is the same as other East African Countries |  |  |
| 2 | Proportion of elderly women in Zanzibar is growing |  |  |
| 3 | Personality changes with ages |  |  |
| 4 | Elderly person’s height tends to decline |  |  |
| 5 | The five senses (sight, hearing, touch, smell and taste) all tend to weaken in old age |  |  |
| 6 | The bladder capacity decreases with age, which leads to frequent urination. |  |  |
| 7 | As compared to younger persons, the elderly (60+yrs) are limited in their activities more often because of suffering from chronic diseases |  |  |
| 8 | As people live longer they face fewer acute conditions and more chronic health conditions |  |  |
| 9 | The majority of old people (past 65 years) have Alzheimer’s disease. |  |  |
| 10 | The Lung capacity of an elderly person is just the same as during childhood |  |  |
| 11 | The majority of old people have no interest in, nor capacity for sexual relations. |  |  |
| 12 | Physical strength tends to decline in old age. |  |  |
| 13 | The size of the liver decreases after the age of 60 years |  |  |
| 14 | Compared to young people, cardiac output and recovery time among the elderly is lower and slower |  |  |
| 15 | Compared to young people, subcutaneous tissue and elastic fibers among elderly tend to increase |  |  |
| 16 | Compared to person under 60, sleep patterns among the elderly tend to decrease steadily |  |  |
| 17 | Elderly persons are have more injuries at home as compared to young ones |  |  |
| 18 | In Zanzibar, the income gap between the elderly and other adult groups continues to widen, whereas the elderly are the poorest group |  |  |
| 19 | As a healthy person reaches old age, his or her voluntary participation in organizations usually decline vividly |  |  |
| 20 | Compared to persons under age 60, rates of criminal activities victimization among the elderly increases steadly |  |  |
| 21 | Compared to younger persons, the elderly persons have more fear of crime |  |  |
| 22 | Sickness is a part of normal aging process |  |  |
| 23 | Regarding blood pressure of an elderly person, systolic blood pressure tend to rise due to loss of elasticity of arteries |  |  |
| 24 | Jean Piaget theory explains the psychological development aspect “Integrity Versus Despair” |  |  |
| 25 | Alcoholism is a very big problem among elderly |  |  |

**Attitudes towards older people measured by Kogan’s Older people Scale. (34 statements)**

**Rating: 1 = Strong disagreed; 2 = disagreed; 3 = Neutral; 4 = Agreed; and 5 = strongly agreed. (Tick appropriate box)**

| S/N |  | 1 | 2 | 3 | 4 | 5 |
| --- | --- | --- | --- | --- | --- | --- |
| 1 | It will be better if most elderly people lived in housing with people of their own age |  |  |  |  |  |
| 2 | It would be better if most elderly people lived in housing that also live young people |  |  |  |  |  |
| 3 | There is something different about most elderly people: It is hard to figure out what makes them different |  |  |  |  |  |
| 4 | Most elderly people are not different from anybody else |  |  |  |  |  |
| 5 | Most elderly people are set in their ways and unable to change |  |  |  |  |  |
| 6 | Most elderly people are capable of new adjustment when the situation demands it |  |  |  |  |  |
| 7 | People grow wiser with old age |  |  |  |  |  |
| 8 | It is foolish to claim that wisdom comes with old age |  |  |  |  |  |
| 9 | Most elderly people bore others by talking about the “good old days” |  |  |  |  |  |
| 10 | Most elderly people past experiences are interesting |  |  |  |  |  |
| 11 | Elderly people are really interesting to nurse |  |  |  |  |  |
| 12 | Nurse work with older people because they cannot cope with high technology care |  |  |  |  |  |
| 13 | Once you will work with older people it is difficult to get job elsewhere |  |  |  |  |  |
| 14 | Are you willing to take care of the elderly people age 60 or over as career choice |  |  |  |  |  |
| 15 | The older you are the easier it is to have good rapport with older people |  |  |  |  |  |
| 16 | Most elderly people seldom complain about the young generations behavior |  |  |  |  |  |
| 17 | Most old people need no more love and reassurance than anyone else. |  |  |  |  |  |
| 18 | Most elderly people make excessive demands for love and reassurance |  |  |  |  |  |
| 19 | A nice neighborhood is the one that has number old people living in it |  |  |  |  |  |
| 20 | You can count on finding a nice residential neighbourhood when there is a sizeable number of old people living in it. |  |  |  |  |  |
| 21 | Most old people are irritable, grouchy, and unpleasant. |  |  |  |  |  |
| 22 | Most old people are cheerful, agreeable, and good humoured |  |  |  |  |  |
| 23 | Most old people like interfering into the affairs of others |  |  |  |  |  |
| 24 | Most old people should have more power in society |  |  |  |  |  |
| 25 | Most old people are constantly complaining about the behaviour of the younger generation. |  |  |  |  |  |
| 26 | One seldom hears old people complaining about the behaviour of the younger generation. |  |  |  |  |  |
| 27 | Most old people should be more concerned with their personal appearance; they’re too untidy. |  |  |  |  |  |
| 28 | Most old people seem quite clean and neat in their personal appearance |  |  |  |  |  |
| 29 | Most older people make one feel ill at ease. |  |  |  |  |  |
| 30 | Most older people are very relaxing to be with |  |  |  |  |  |
| 31 | There is a need to promote by routine health checkups, elderly health camps and health education sessions |  |  |  |  |  |
| 32 | It will be interesting to work in a special ward for elderly rather than mix them with others |  |  |  |  |  |
| 33 | Most old people should have more power in society |  |  |  |  |  |
| 34 | Most old people like interfering into the affairs of others |  |  |  |  |  |
